# Highly Soluble β-Glucan Fiber Modulates Mechanisms of Blood Glucose Regulation and Intestinal Permeability

**DOI:** 10.3390/nu16142240

**Published:** 2024-07-12

**Authors:** Angela M. Marcobal, Bruce R. McConnell, Riley A. Drexler, Katharine M. Ng, Maria X. Maldonado-Gomez, Alexandria M. S. Conner, Cory G. Vierra, Nithya Krishnakumar, Hannah M. Gerber, Jada K. A. Garcia, James P. Cerney, Matthew J. Amicucci

**Affiliations:** One Bio Inc., Sacramento, CA 95833, USA

**Keywords:** β-glucan fiber, oligosaccharides, hyperglycemia, highly soluble fiber

## Abstract

β-glucans found in cereal grains have been previously demonstrated to improve blood glucose control; however, current understanding points to their high viscosity as the primary mechanism of action. In this work, we present a novel, highly soluble, low-viscosity β-glucan fiber (HS-BG fiber) and a preclinical dataset that demonstrates its impact on two mechanisms related to the prevention of hyperglycemia. Our results show that HS-BG inhibits the activity of two key proteins involved in glucose metabolism, the α-glucosidase enzyme and the SGLT1 transporter, thereby having the potential to slow starch digestion and subsequent glucose uptake. Furthermore, we demonstrate in a multi-donor fecal fermentation model that HS-BG is metabolized by several different members of the gut microbiome, producing high amounts of short-chain fatty acids (SCFAs), known agonists of GPR43 receptors in the gut related to GLP-1 secretion. The production of SCFAs was verified in the translational gut model, SHIME^®^. Moreover, HS-BG fiber fermentation produces compounds that restored permeability in disrupted epithelial cells, decreased inflammatory chemokines (CXCL10, MCP-1, and IL-8), and increased anti-inflammatory marker (IL-10), which could improve insulin resistance. Together, these data suggest that the novel HS-BG fiber is a promising new functional ingredient that can be used to modulate postprandial glycemic responses while the high solubility and low viscosity enable easy formulation in both beverage and solid food matrices.

## 1. Introduction

Cereal β-glucans are natural polysaccharides found in cereal grains such as barley, oat, rye, or wheat. Cereal β-glucans are linear polysaccharides composed of glucose bound through β-1,4 and β-1,3 linkages and are associated with many health benefits [1]. The United States Food and Drug Administration (FDA) has approved the use of a health claim for the impact of cereal β-glucan in reducing the risk of coronary disease [2]. Moreover, the European Food Safety Authority (ESFA) has approved health claims for cereal β-glucans related to a reduction in postprandial glycemic responses and serum cholesterol [3,4]. Additional studies suggest that ingestion of cereal β-glucan has other benefits such as glycemic control in type 1 diabetes [5], attenuating cognitive impairment [6], improving chronic kidney diseases [7], and reducing fatigue [8].

The International Diabetes Federation reports that, in 2021, about 537 million adult people were living with diabetes. They project this to increase up to 783 million by 2045. The economic cost of this is estimated to be USD 412.9 billion in the US alone [9]. Prolonged hyperglycemia can lead to serious damage to many of the body’s systems. For example, it is associated with an increased risk of microvascular complications such as retinopathy, neuropathy, and nephropathy, and macrovascular complications such as myocardial infarction, cardiovascular disease, and stroke [10]. Further, it contributes to the progressive worsening of insulin resistance, fueling a continued cycle of metabolic decline [11] and diabetes. Consequently, achieving glycemic control is the key principle in diabetes management, including reducing the risk of developing type 2 diabetes. The gold standard for assessing glycemic control is A1C, and treatment guidelines for diabetes have historically focused on reducing A1C to specified targets [12]. However, A1C levels are the result of a combination of both fasting blood glucose (FBG) and postprandial blood glucose (PPBG) levels [13]. Therefore, long-term A1C target levels cannot be achieved by targeting FBG only. PPBG must also be targeted and most treatment guidelines now include specific PPBG targets [14]. Despite this, current therapies (e.g., metformin, sulfonylureas, thiazolidinediones, and basal insulins) are mainly effective in controlling FBG, and PPBG has been given less attention [9].

PPBG levels are governed by insulin production and glucose absorption in the small intestine and are influenced by diverse environmental factors such as diet and the intestinal microbiota. The highly processed Western diet, with high amounts of sugar and starch and a paucity of dietary fiber, affects diverse mechanisms related to glucose absorption in our bodies, accelerating the development of metabolic disorders [15]. Dietary supplementation with cereal β-glucans has been suggested as a safe, long-term approach to managing PPBG, as these fibers have known PPBG-reducing properties and can partially compensate for the existing fiber gap in the Western diet.

The blood-glucose-lowering effect of cereal β-glucan has primarily been attributed to its ability to increase viscosity in the small intestine [16], which is believed to delay gastric emptying and reduce the absorption of glucose by the host [3,17]. This ability to increase viscosity is a characteristic rheological property of all cereal β-glucans currently on the market [18]. Additional mechanisms beyond viscosity have been described for β-glucan, but to a lesser extent. β-glucans can impact the host and gut microbiome by inhibiting key digestive enzymes and glucose transporters [17,19] and stimulating GLP-1 production through butyrate, a product of carbohydrate fermentation by gut microorganisms [20].

To make a postprandial glycemic reduction claim, the amount of β-glucan that is required to be added to a product is relatively high. For instance, ESFA requires at least 4 g of cereal β-glucan per 30 g of available carbohydrates, which is roughly the ratio of β-glucan to carbohydrates in 50 g of uncooked oats [3]. The need to deliver this relatively high dose of β-glucan is therefore achieved by either integrating large amounts of oat or barley into food products or by extracting the cereal β-glucans for their integration, both of which create issues in food processing and negatively impact organoleptic properties and sensory qualities [21]. This is particularly the case for liquid products such as beverages, where the high viscosity of even extracted cereal β-glucans results in poor consumer experiences. Therefore, although β-glucan is a promising functional ingredient, its integration into food and beverages at higher doses remains a challenge, which has limited its use in the food industry. A possible solution is to reduce the molecular weight and viscosity of the β-glucan. However, due to a lack of availability, the question remains of whether soluble, non-viscous β-glucans, which have a sufficiently lower degree of polymerization, maintain the health benefits of the native fiber.

In this work, we aim to evaluate the impact of a novel, highly soluble, non-viscous, β-glucan (HS-BG) fiber on mechanisms related to blood glucose control. HS-BG fiber is the product of the Fenton-induced depolymerization of oat β-glucans [22]. Fenton chemistry can be used as a universal depolymerizer of polysaccharides, thereby lowering the molecular weight, between 0.5 and 50 kDa in the case of HS-BG, and increasing the solubility of fibers. When controlled properly, Fenton chemistry induces cleavage of the glycosidic bonds between monosaccharide residues without altering the monosaccharide profile or glycosidic linkage composition, and unlike other forms of grain fiber processing, it limits the formation of oxidative side products [23]. The presence of oxidized hydroxyl groups along the chain has been demonstrated to change the physicochemical properties of fiber during food processing but is not commonly discussed as a cause for concern in relation to human health. It also has a pleasant taste, enabling the inclusion of high doses of β-glucan in beverages and a wide array of food products, a feature that cereal β-glucans traditionally struggle to do. Testing of the HS-BG fiber in different in vitro models demonstrates that HS-BG fiber modulates several key mechanisms for modulating the postprandial glycemic response. These results suggest that ingestion of low-molecular-weight β-glucan can have an important role in blood glucose control.

## 2. Materials and Methods

The HS-BG fiber used in the studies was provided by One Bio Inc. The material was produced using oat fiber ingredients as a starting material (Huzhou Purestar Biochem, Huzhou, China). HS-BG fiber is highly enriched in oligosaccharides (>70%), which are composed of linear, mixed-linkage (1-3), (1-4)-β-D-glucans.

### 2.1. Digestive Enzyme Inhibition Assay

The inhibition of α-glucosidases by HS-BG fiber was determined by incubating α-glucosidase (2 U/mL, >99% purity, Creative Biomart Therapeutic Proteins, Shirley, NY, USA) in the presence of maltose (20 μM, 95% purity, Bean Town Chemicals, Hudson, NH, USA) and HS-BG fiber (10 mg/mL) and periodically monitoring the concentrations of maltose (substrate) and free glucose (product) throughout the reaction. Acarbose (200 μM, ≥94% purity, Thermo Scientific Chemicals, Waltham, MA, USA) was used as a positive inhibitory control. Three tubes per treatment were prepared and incubated at 37 °C. The progression of the reaction was captured at 0, 10, 30, and 60 min by placing tubes in a 96 °C water bath for 10 min to inactivate the enzyme. Glucose and maltose were measured in a modified manner by Xu et al. [24]. Briefly, glucose and maltose were derivatized with PMP (3-Methyl-1-phenyl-2-pyrazoline-5-one, Sigma Aldrich, St. Louis, MO, USA) and analyzed on a UHPLC/QqQ mass spectrometer (Agilent Technologies, Santa Clara, CA, USA). Quantitation was performed by employing a standard curve, and the peak area was normalized by employing internal standards.

### 2.2. SGLT1 Inhibition Assay

An SGLT1 inhibition assay was performed at Charles River Laboratory (Solvo Biotechnology, Budapest, Hungary), according to their internal protocol, based on Kanwal et al.’s studies [25]. Briefly, HS-BG fiber was diluted in the appropriate assay buffer at the final tested concentration and pre-warmed separately at 37 °C for 15 min. Two different cell lines were used in the assay: HEK293 cells expressing SGLT1 and the respective control cell lines (HEK293-Mock-Fin). Prior to the experiment, the cell culture medium was removed, and cells were rinsed with an assay buffer. Transporter-expressing and control cells were pre-incubated at 37 °C with an assay buffer. Alpha-methyl glucoside (AMG) at a concentration of 1 μM served as a probe substrate, and four different concentrations of HS-BG fiber were tested in triplicate (2.1 mg/mL, 6.8 mg/mL, 9.8 mg/mL, and 12.7 mg/mL). Phlorizin (100 μM) was used as a positive control, being the reference inhibitor to ensure maximal inhibition of SGLT1. Following a 30 min incubation, cells were rinsed twice with the assay buffer and lysed to assess substrate uptake. The amount of substrate in the lysate was determined by liquid scintillation counting.

### 2.3. Static Fecal Fermentations

Static fecal fermentations were conducted in a deep 96-well format, based on a previously described method with minor modifications [26]. Fecal fermentations were run under anaerobic conditions (Anaerobic Chamber Vinyl Type B, Coy Labs, Grass Lake, MI, USA), using a mix of gas (carbon dioxide 5%, hydrogen 5%, nitrogen balance, Linde, Danbury, CT, USA). Fermentation media were optimized to support diverse microbial taxa and control pH within the range of the colon’s physiological conditions, containing mineral and vitamin solution, CaCl_2_ (10 mg/mL), and a basic fermentation medium [27]. A mix of background sugars (xylan, amylopectin, potato starch, and pectin) was included in the basic fermentation media. Fermentation was performed using 10 fecal samples from healthy individual donors (2% of the fermentation media). The effect of HS-BG fiber on microbial communities was assessed at a concentration of 0.6% *w*/*v*. Similar fermentations were run without added oligosaccharides (untreated controls). Four replicates were run for each condition. Samples were taken at multiple time points (0 h, 10 h, and 24 h) for short-chain fatty acid (SCFA) analysis. Briefly, fecal supernatants were derivatized with 3-nitrophenylhydrazine (3-NPH) for LC-MS/QqQ analysis. Analysis was performed using a 1290 Infinity II LC (Agilent Technologies, USA) equipped with a reverse phase column (Zorbax Eclipse C18 2.1 × 50 mm; Agilent Technologies, USA) and a 6490 Triple Quad LC/MS (Agilent Technologies, USA). SCFAs were analyzed based on a previously described method [28], with a 1290 Infinity II LC (Agilent Technologies, USA) equipped with a reverse phase column (Zorbax Eclipse C18 2.1 × 50 mm; Agilent Technologies, USA) and a 6490 Triple Quad LC/MS (Agilent Technologies, USA). LC separation was performed with 5% acetonitrile with 0.1% formic acid (solvent A) and 95% acetonitrile with 0.1% formic acid (solvent B). Peak areas were quantitated using Agilent Quantitative Analysis software v10.1, and areas were normalized to internal standards and compared to an external standard curve for quantitation.

In addition, after 24 h of fermentation, gDNA was extracted from each well and sent for 16S rRNA sequencing. DNA was extracted from fecal slurries using the ZymoBIOMICS Kit D4308 (Zymo Research, Tustin, CA, USA) and a KingFisher Flex DNA extraction robot (Thermo Fisher Scientific, USA). Microbial communities were profiled by sequencing the V4 region of the bacterial 16S rRNA gene amplified using 515F (5′-GTGCCAGCMGCCGCGGTAA-3′) and 806R (5′ GGACTACHVGGGTWTCTAAT-3′) primers. NovaSeq 6000 (Illumina, San Diego, CA, USA) was used to obtain 250 bp paired-end reads. Raw demultiplexed reads were processed using QIIME2 2020.11 [29]. Briefly, after quality checking, trimming, filtering, and denoising were performed using the “dada2 denoise-paired” plugin in QIIME2. A taxonomic classification of ASVs was performed with the “q2-feature-classifier” plugin and a naive Bayes classifier trained on SILVA 138 99% OTUs from the 515F/806R region of 16S rRNA sequences.

### 2.4. Mucosal Simulator of Human Intestinal Ecosystem (SHIME^®^)

An M-SHIME^®^ study was performed by ProDigest (Gent, Belgium) through a modification of the technology previously described [30]. This system simulates the physiology and microbiology of the gastrointestinal tract. The reactor setup consists of a succession of three reactors. The first one simulates stomach/small intestine conditions (ST/SI), and the other two simulate the large intestine (proximal colon (PC) and distal colon (DC)). The PC and DC vessels were inoculated with a selected fecal sample. The ST/SI vessel was operated according to a fill and draw principle, and the PC and DC reactors had a fixed volume of 500 and 800 mL, respectively. The pH of the PC and DC was controlled using 0.5 M NaOH and 0.5 M HCl. To simulate both the luminal and mucus-associated microbial community, a mucosal compartment was included in the colon reactors by the addition of mucin-coated beads (AnoxKaldes, Lund, Sweden). All reactors were airtight, continuously agitated (300 rpm), and maintained at 37 °C. Anaerobiosis was maintained by flushing the headspace of all reactors once per day with nitrogen gas (CompAir Geveke, Vilvoorde, Belgium). The experiment was divided into three different stages: a control stage (2 weeks, a stabilization of reactors and a fecal sample, being the baseline microbial community and activity), a treatment stage (3 weeks, in which HS-BG fiber was added daily), and a washout stage (2 weeks, to test the permanence of the effect after an interruption of the treatment). During the treatment stage, HS-BG fiber was administered to an equivalent dose in humans of 6 g/day.

ProDigest collected samples 3 times per week during each phase of the experiment to determine microbial activity by measuring levels of SCFAs. SCFAs were quantified by gas chromatography (GC 2014-AOC20i autosampler, Shimadzu Europa GmBH, Bruxelles, Belgium) [31]. ProDigest collected luminal and mucin samples at the end of the control, treatment, and washout phases to determine changes in microbial composition by 16S rRNA sequencing. gDNA was extracted and sequenced as described for static fermentations.

### 2.5. Measurement of Intestine Epithelial Barrier Integrity and Inflammatory Markers

Co-cultures of Caco-2 and THP-1 cells were used to assess the protective effect of the SHIME^®^ colonic suspensions, resulting from the fermentation of HS-BG fiber. This co-culture model was performed at ProDigest (Belgium), following methods described previously [32]. Caco-2 cells (HTB-37; ATCC) were seeded in 24-well semi-permeable inserts. Caco-2 monolayers were cultured for 14 days, with 3 medium changes/week, until a functional monolayer with a transepithelial electrical resistance (TEER) of more than 300 cm^2^ was obtained (measured with a Millicell ERS-2 epithelial volt-/ohmmeter, Millipore, Burlington, MA, USA). The TEER of the Caco-2 monolayers was measured (0 h time point), and this value was subtracted from all readings to account for the residual electrical resistance of an insert.

THP1-Blue™ monocytes, isolated from a human patient with acute leukemia, were activated upon phorbol 12-myristate 13-acetate (PMA) treatment. Then, the Caco-2-bearing inserts were placed on top of the PMA-differentiated THP1-Blue™ cells. When Caco-2 cells are seeded onto trans-well plates and placed on top of PMA-activated THP-1 cells, their monolayer becomes disrupted (measured as a decrease in the TEER value).

The apical compartment (containing the Caco-2 cells) was filled with sterile-filtered (0.22 μm) colonic suspensions from the SHIME^®^ experiment, diluted 1:5 *v*/*v* in a complete medium (CM). The basolateral compartment (containing the THP1-Blue™ cells) was filled with Caco-2 complete medium (CM). Cells were also exposed to a CM in both chambers as the negative control. In addition, a positive control was added by filling the apical compartment with sodium butyrate (Sigma-Aldrich, USA). TEER values were measured after 24 hrs. After subtracting the TEER of the empty insert, all 24 h values were normalized to their own 0 h value and are presented as a percentage of the initial value.

The basolateral supernatant was discarded after the TEER measurement, and cells were stimulated at the basolateral side with CM containing ultrapure LPS. As a control, hydrocortisone (HC) was added as a broad immunosuppressant of LPS stimulation. Six hours after LPS stimulation, cytokines (IL-1β, IL-10, IL-6, and TNF-α) and chemokines (CXCL10, MCP-1, and IL-8) were measured by the Luminex^®^ multiplex (Thermo Fisher Scientific, USA).

### 2.6. Statistical Analysis

All figures and statistical analyses were performed using R Statistical Software (v4.3.0; R Core Team 2023). The figures were created using the ggplot2 R package (v3.4.2; Wickham 2016) and the ggprism R package (v1.0.4; Dawson 2022). Paired and unpaired *t*-tests were calculated and plotted using the rstatix R package (v0.7.2; Kassambara 2023). Calculated *p*-values greater than 0.05 were considered not significant (ns).

For the digestive enzyme inhibition assay, 3 replicates per time point were run for each treatment. Averages of replicates are plotted as points with standard deviations as error bars. *t*-tests comparing the abundance of glucose or maltose at the 60 min time point were performed using the HS-BG treatment as a reference group.

For the SGLT1 inhibition assay, 3 replicates per tested concentration were run. The relative inhibition for each replicate was calculated by subtracting the relative transporter-specific accumulation for a given sample (%) from the relative transporter-specific accumulation for the assay buffer without fiber (%). Replicates were averaged and standard deviations were calculated. Data were provided by Charles River Laboratory.

For the static fecal fermentation assay, 3 replicates were run for each of the 10 fecal donors. Net SCFA production was calculated for each replicate by subtracting the 0 h value from the 24 h value for each analyte. This was carried out to remove any baseline readings coming from the fecal sample. Pairwise *t*-tests within donors were used to evaluate the effect of HS-BG for each analyte. *p*-values were corrected for multiple comparisons using the Benjamini–Hochberg method. Relative abundances of butyrate producers were compared using pairwise *t*-tests within donors, and *p*-values were corrected for multiple comparisons using the Benjamini–Hochberg method.

For the SHIME^®^ assay, ProDigest collected samples 3 times per week during each phase of the experiment to determine microbial activity by measuring levels of SCFAs. The percent increase from the control period for each analyte was calculated by using the final sample taken during the last week of the control period as the reference for change. ProDigest collected luminal and mucin samples at the end of each phase to determine changes in microbial composition by 16S rRNA sequencing. The 16S rRNA sequencing data are reported in relative abundance. Data were provided by ProDigest.

For the measurement of intestine epithelial barrier integrity and inflammatory markers, 3 replicates were run for the control phase and the treatment with HS-BG phase. *t*-tests were performed within each colonic vessel group for each immune marker. Data were provided by ProDigest.

## 3. Results

### 3.1. HS-BG Fiber Inhibits α-Glucosidase Enzymatic Activity

**Figure 1:**
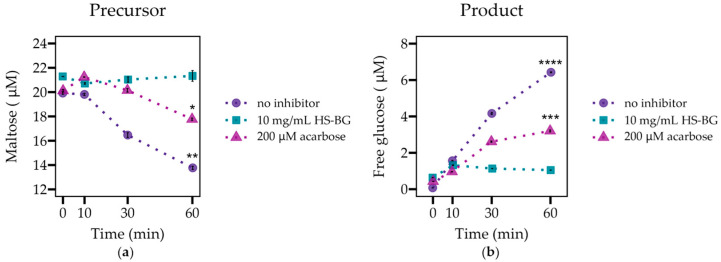
The evaluation of α-glucosidase activity (*n* = 3) as shown by (**a**) maltose degradation and (**b**) glucose generation, when incubated with maltose in the presence and absence of 10 mg/mL of HS-BG fiber. Acarbose 200 µM served as a positive control of inhibition (**** *p* ≤ 0.0001, *** *p* ≤ 0.001, ** *p* ≤ 0.01, * *p* ≤ 0.05).

The digestive enzyme α-glucosidase is localized in the brush border of the small intestine and is responsible for the enzymatic hydrolysis of α-1,4-linked disaccharides, oligosaccharides, and polysaccharides found in many starchy foods, including potatoes, rice, cereal grains, and corn, producing absorbable monosaccharides. The release of free glucose due to the activity of this enzyme contributes to hyperglycemia in some individuals [33]. Therefore, the inhibition of this enzyme is a target for the modulation of postprandial glucose spikes. We performed an experiment to evaluate the inhibition of α-glucosidase during incubation with the α-1,4-linked disaccharide, maltose (20 µM), in the presence of HS-BG fiber (10 mg/mL). We compared the results with a positive control inhibitor (200 µM acarbose) in the absence of the fiber. We did not include a long-chain β-glucan for comparison due to the high viscosity in the solution. When α-glucosidase was incubated for 60 min in the presence of maltose alone, maltose levels decreased and free glucose accumulated, as expected (Figure 1). When HS-BG fiber was added to the reaction media, maltose degradation was nearly 100% inhibited. In the presence of the fiber, glucose levels were almost constant, with an increase of just 15% during incubation with the enzyme. Acarbose, at the assayed concentration, partially inhibited the activity of the α-glucosidase, and the measured glucose production was 52% compared to glucose produced in the no-inhibitor control. This result supports the hypothesis that HS-BG fiber inhibits the activity of α-glucosidase.

### 3.2. HS-BG Fiber Inhibits Intestinal Glucose Transporter SGLT1

The high-affinity low-capacity sodium/glucose cotransporter, SGLT1, is responsible for the active transport of glucose across the brush border membrane of the small intestine, a key process in the postprandial increase in blood glucose. To measure the potential of HS-BG fiber to inhibit SGLT1 activity, we used a cellular uptake assay, in which HEK293 cells expressing SGLT1 were exposed to a probe substrate, α-methyl glucoside (AMG), and different concentrations of HS-BG fiber. When selecting the amounts of HS-BG fiber to be tested, we evaluated concentrations up to 12.7 mg/mL, which corresponds to a concentration that could be used in a beverage product labeled as a “good source” of fiber (21CFR101.5418). As before, we could not include the native long-chain β-glucans for comparison since their high viscosity made it impossible to run this cell-based assay. Using phlorizin as a positive control for 100% activity, we measured the relative transporter accumulation of AMG and determined relative enzyme inhibition. Concentrations of 2.1 mg/mL HS-BG resulted in a 6% inhibition of SGLT1 activity. We observed a dose-dependent increase in SGLT1 with increasing concentrations of HS-BG fiber; 6.8 mg/mL inhibited SGLT1 activity by 12%, 9.8 mg/mL inhibited SGLT1 activity by 17%, and 12.7 mg/mL inhibited activity by 23% (Table 1). Our results demonstrate that HS-BG fiber inhibits human SGLT1 in a dose-dependent manner.

### 3.3. HS-BG Fiber Consistently Modulates Microbial Composition and Butyrate Production in Static Fecal Fermentations across Multiple Fecal Donors

**Figure 2:**
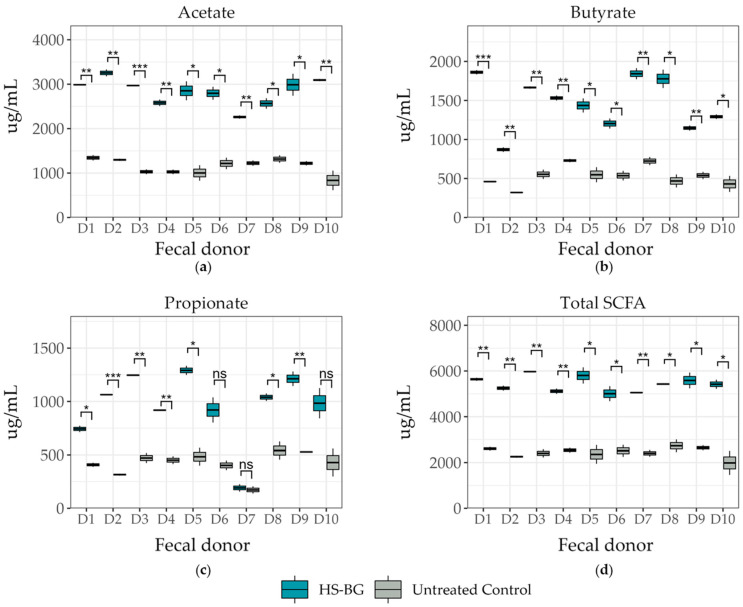
Short-chain fatty acid production after 24 h fecal fermentation (10 donor samples, *n* = 3) for metabolites of (**a**) acetate, (**b**) butyrate, and (**c**) propionate, and (**d**) total SCFA production (*** *p* ≤ 0.001, ** *p* ≤ 0.01, * *p* ≤ 0.05, not significant (ns) *p* > 0.05).

β-glucan is a known growth substrate for a range of beneficial microorganisms found in the intestinal microbiota. The fermentation of β-glucan by these microorganisms can lead to the production of short-chain fatty acids (SCFAs), an important class of compounds with multiple beneficial effects on human health. One of the most important roles that SCFAs play is as agonists of GPR43 receptors; their activation results in the stimulation of glucagon-like peptide-1 (GLP-1) secretion, which has an important role in blood glucose regulation. To determine the impact of HS-BG fiber on the metabolic profile and composition of the gut microbiota, fecal samples from a population of 10 healthy donors were assayed in a high-throughput static fecal fermentation experiment. We observed a significant decrease in pH after 24 h incubation under anaerobic conditions, suggesting a high degree of fermentability of the fiber by the gut microbial communities. An analysis of growth supernatants after 24 h by LC-MS/QqQ confirmed a high amount of SCFAs in samples fermented in the presence of HS-BG fiber. Fecal fermentations supplemented with HS-BG fiber produced significantly higher amounts of SCFAs than microbial fecal fermentations in the absence of fiber. Specifically, butyrate, acetate, and propionate production were significantly increased by HS-BG metabolism (Figure 2). All tested donors show a similar fermentation product profile, indicating a consistent production of beneficial metabolites that overcome differences in each unique microbiota composition.

**Figure 3:**
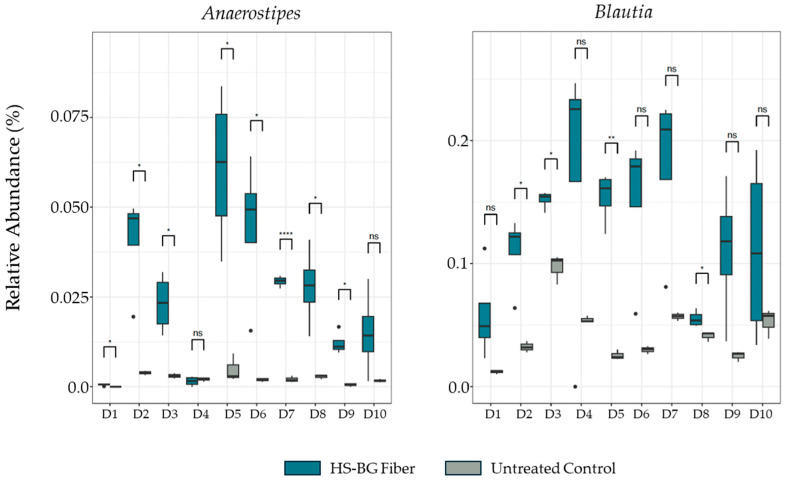
Changes in relative abundance of butyrate producers *Anaerostipes* and *Blautia* after 24 h fecal fermentation (10 donor samples, *n* = 3). **** *p* ≤ 0.0001, ** *p* ≤ 0.01, * *p* ≤ 0.05, not significant (ns) *p* > 0.05.

The increase in butyrate and other SCFAs after 24 h fermentation was accompanied by a concomitant and significant increase in specific taxa related to butyrate production relative to the untreated control. Specifically, in HS-BG fiber-supplemented fermentations, we observed an increase in the relative abundance of *Anaerostipes* and *Blautia* across most of the tested population (Figure 3). While in some donors this increase was not statistically significant, we observed a common trend across the population that suggested that HS-BG fiber supplementation significantly impacted the production of butyrate.

**Figure 4:**
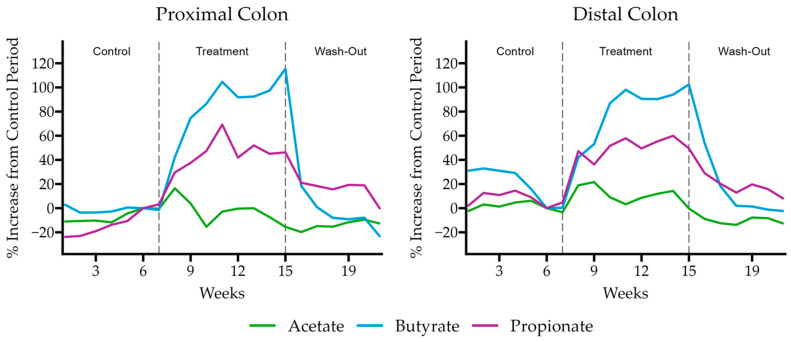
Butyrate, propionate, and acetate production relative to the control period of the SHIME model experiment when supplemented with HS-BG fiber (*n* = 1).

We confirmed these results in a multi-compartment dynamic simulator of the human gut, the Simulator of the Human Intestinal Microbial Ecosystem (SHIME^®^) model. In this model, the retention time and pH of the vessels were chosen to resemble in vivo conditions in the proximal and distal colon. After an initial control period of 2 weeks, a 3-week treatment period with HS-BG supplementation was performed, with concentrations chosen to mimic a human dose of 6 g/day. To gain insight into the overall changes in microbial activity, butyrate, propionate, and acetate were measured throughout the experiment at various time points (Figure 4). We observed a significant increase in butyrate during the treatment phase. By the end of the treatment phase, butyrate increased by more than 100% from the control phase (115% in the proximal and 102% in the distal colon). In addition, propionate production significantly increased compared to the control period (46% proximal, 50% distal). The levels of acetate fluctuated during HS-BG fiber exposure, but they did not increase significantly at the end of the treatment phase. After cessation of the treatment, the concentrations of these SCFAs returned to their pretreatment levels.

**Figure 5:**
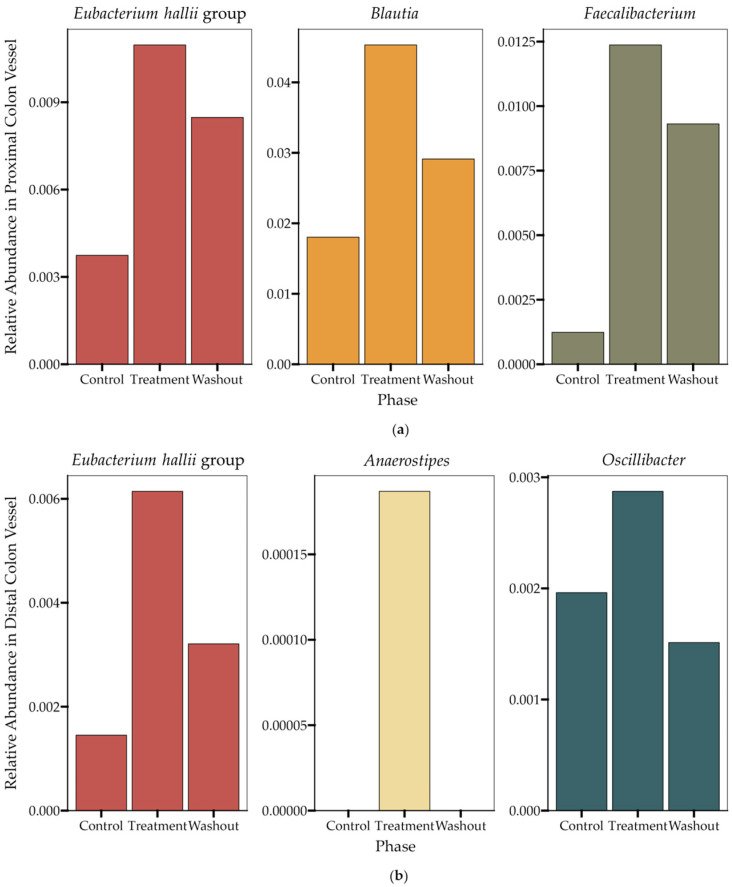
Changes in the relative abundance of butyrate-producing taxa (*n* = 1) during the SHIME^®^ experiment in (**a**) the proximal colon vessel and (**b**) the distal colon vessel. The data displayed represent the final time points taken during the last week in each of the three phases (control, treatment, washout).

A 16S RNA sequencing analysis of the microbial composition in the proximal and distal colon vessels before and during treatment demonstrated the impact of HS-BG fiber impact on specific butyrate-producing microbial taxa. In the proximal colon vessel, we observed an increase in the relative abundance of the butyrate producers *Eubacterium hallii* group, *Blautia* and *Faecalibacterium,* during the treatment period with HS-BG fiber, and in the distal colon vessel, we observed increases in the butyrate-producing taxa *Eubacterium hallii* group, *Anaerostipes* and *Oscillibacter* (Figure 5). This result aligns with the increase in butyrate-producing taxa and the production of high levels of butyrate observed in the static fecal fermentations (Figure 2). Interestingly, butyrate producers demonstrated different dynamics post-treatment. *Eubacterium hallii* remained at a high relative abundance after the treatment phase was concluded; however, with other butyrate-producing taxa such as *Blautia* and *Faecalibacterium*, their higher abundance was supported by HS-BG fiber supplementation but decreased during the washout period. Based on the microbial compositional changes and metabolite production in both static fecal fermentations and the SHIME^®^ experiment, we conclude that HS-BG fiber is highly fermentable by the gut microbiome, resulting in robust SCFA production, which may act as an agent for stimulating GLP-1 production and potentially impacting blood glucose levels.

### 3.4. HS-BG Fiber Colonic Suspensions Restore Gut Epithelial Barrier

**Figure 6:**
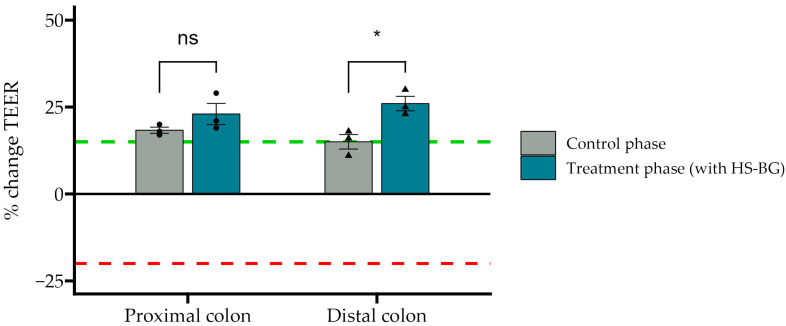
The effect of SHIME colonic suspensions before and after the treatment phase on the TEER values of the Caco-2/THP1-Blue co-cultures. TEER was measured 24 h after incubation with co-cultures. Each 24 h value was compared to its corresponding 0 h value. A value of 0% represents the initial value; the red dotted line corresponds to a CM without SHIME^®^ colonic suspensions, and the green dotted line corresponds to NaB applied to the SHIME^®^ colonic suspensions (* *p* ≤ 0.05, not significant (ns) *p* > 0.05).

The healthy gut epithelial barrier plays an important and active role in glucose absorption and glycemic control, in addition to many other aspects of human health. When the gut barrier is healthy and tight junctions between epithelial cells are strong, glucose absorption is effective, and macromolecules that may cause inflammation and insulin resistance are not absorbed [34]. TEER measures the “tightness” of tight junctions, and a reduction in the TEER value reveals a loss of barrier function. Using colonic suspensions from the described SHIME^®^ experiment and Caco2 cell cultures, we examined the effect of HS-BG fiber fermentation on the function of the gut epithelial barrier. Colonic suspensions from SHIME^®^ proximal and distal colon vessels were collected before and after the treatment period, diluted in a CM, and given apically to the co-cultures for 24 h. The TEER results demonstrated that supernatants from fermentations of HS-BG fiber in the proximal and distal colon vessels were able to maintain and even further increase the TEER values compared to their initial values (*p* = 0.02) (Figure 6). The positive control sodium butyrate behaved as expected, increasing the TEER value.

**Figure 7:**
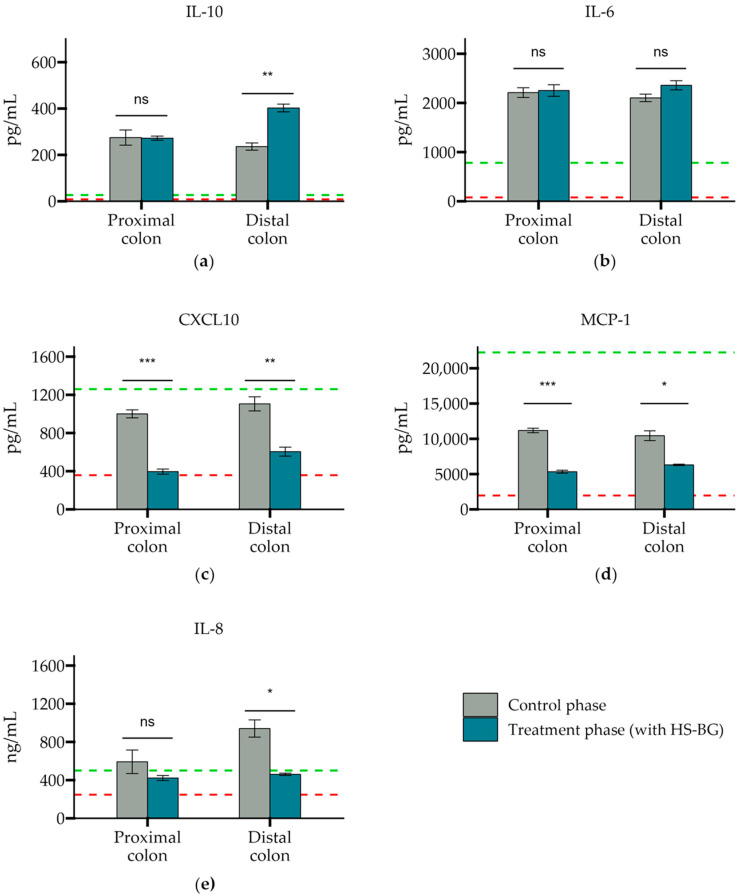
The secretion of cytokines and chemokines (**a**) IL-10, (**b**) IL-6, (**c**) CXCL10, (**d**) MCP-1, and (**e**) IL-8 after exposure to a SHIME colonic suspension on LPS-stimulated THP-1 cells. Cytokine levels were measured 6 h after LPS treatment on the basolateral side of the Caco-2/THP1-Blue™ co-cultures after pre-treatment of the apical side for 24 h with SHIME suspensions. The red dotted line corresponds to the experimental control LPS, and the green dotted line corresponds to the experimental control LPS + HC (*** *p* ≤ 0.001, ** *p* ≤ 0.01, * *p* ≤ 0.05, not significant (ns) *p* > 0.05).

In addition, to assess the effect on inflammation of the epithelial barrier, several immune markers were evaluated, using similar SHIME colonic suspensions as above, from proximal and distal colon vessels before and after the treatment phase. We observed that all controls behaved as expected in this experiment. LPS was able to increase the secretion of anti-inflammatory cytokine IL-10 and pro-inflammatory chemokines CXCL10, MCP-1, and IL-8. Also, HC acted as a broad immunosuppressant by dampening LPS-induced cytokines and chemokines. In general, colonic suspensions from proximal and distal colonic vessels after HS-BG fiber treatment exerted an anti-inflammatory effect on disrupted epithelial cells (Figure 7). IL-10, an anti-inflammatory cytokine, was significantly increased when cells were treated with a colonic suspension from the distal colon vessel (*p* = 0.002). There was also an increase in IL-6, which has been demonstrated to have a positive effect on the regeneration of the intestinal epithelium and on wound healing, but significance relative to the control phase was not achieved. A significant decrease in pro-inflammatory chemokines CXCL10 (*p* = 0.001 proximal, *p* = 0.008 distal) and MCP-1 was observed in both proximal and distal colon vessels (*p* < 0.001 proximal, *p* = 0.03 distal), and a significant reduction in IL-8 production was observed in the distal colon vessel (*p* = 0.03) (Figure 7). These results indicate that metabolites present in the supernatants can provide a restorative effect on epithelial cell function, which is crucial in ensuring the adequate function of the intestinal cell wall and potentially blood glucose levels.

## 4. Discussion and Conclusions

The benefits of cereal β-glucans in the management of hyperglycemia have been known for decades, but the use of these fibers remains limited. Until now, most studies that explain the impact of β-glucan on glucose control have been focused on the effect of fiber viscosity on glucose diffusion and subsequent transport [35]. However, β-glucan, which can increase viscosity, has a very large degree of polymerization and is difficult to formulate into palatable, easy-to-use products.

We demonstrated that a novel, extensively depolymerized β-glucan (HS-BG) inhibited two key enzymes related to glucose uptake and was robustly fermented by the gut microbiome into SCFAs, metabolites known for their potent effect on GLP-1 and the maintenance of a healthy gut barrier. These findings support our hypothesis that the viscosity of β-glucan constitutes only one of the modes of action for reducing blood glucose levels, and that extensively depolymerized β-glucan is capable of significantly impacting key mechanisms involved in glucose control. A reduction in the polymerization degree of the original β-glucan fiber may enhance the structural capacity of the fiber to inhibit key enzymes related to glucose uptake. HS-BG is also far more suited to widespread use in products due to its minimal impact on organoleptics.

One of the main processes investigated in this paper and targeted by HS-BG fiber is the initial digestion of dietary carbohydrates by digestive enzymes. Pancreatic α-amylase acts in the duodenum, hydrolyzing α-1-4 linked carbohydrates into smaller fragments, such as sucrose and maltose [36]. α-glucosidase, located in the brush border membrane of the small intestine, cleaves maltose into glucose monosaccharides for intestinal absorption [37]. As expected, there is much interest in discovering the synthetic and natural inhibitors of both digestive enzymes. The antidiabetic drug acarbose inhibits α-glucosidase but has reported gastrointestinal side effects, in particular, flatulence and diarrhea. It has also been demonstrated that some natural molecules present in plants, like flavonoids, tannins, or alkaloids, can act as digestive enzyme inhibitors [38]. Several fibers have also been identified as α-glucosidase inhibitors, such as those from psyllium husk and legumes [39] and β-glucan isolated from yeast or mushrooms, which significantly reduce starch digestibility [40,41]. Like acarbose, these fibers act as competitive inhibitors by nature of their structural similarity to the natural oligosaccharide substrates, which enables them to bind to the enzyme’s active site. The close resemblance of many α-glucosidase inhibitors to di- and oligosaccharides suggests that the suppression of activity can be achieved by the structural features of short β-glucan oligosaccharides, independent of the polymerization degree [42]. In this work, we show for the first time that the inhibitory effect of HS-BG fiber on α-glucosidase can replicate the effects of a low-dose acarbose (Figure 1). We hypothesize that HS-BG fiber interacts non-covalently with the enzymes, interfering with the binding site and reducing catalytic activity.

Another key process in blood glucose regulation is the glucose absorption mediated by SGLT-1 transporters located in the brush border of the small intestine, which transport the glucose released by digestive enzymes from the gut lumen to the intestinal epithelium. Some new diabetes medications target SGLT-1 to delay and reduce the absorption of glucose, especially after meals. It has been reported that several natural ingredients demonstrate an inhibition of this transporter, such as specific polyphenols [43], flavonoids [44], or grain fibers [10], which can influence the postprandial peak glucose response by reducing SGLT-1 activity in both in vitro and animal studies. In this work, we showed that HS-BG fiber impacts SGLT-1 transporter activity in a dose-dependent manner (Table 1). As with α-glucosidase, structural features of β-glucan could act as competitive inhibitors, competitively blocking the transporter from binding and transporting glucose from the lumen. These results indicate that HS-BG fiber is a candidate for SGLT-1 inhibition with a potential for the management of PPBG levels.

In addition to the direct interaction of HS-BG fiber with host enzymes and transporters, we explored its effect on the gut microbiome and the production of SCFAs as a product of carbohydrate fermentation. Prebiotic fibers reach the colon where they are fermented by gut microorganisms and transformed into SCFAs. SCFAs are signaling molecules that can bind to the G-protein-coupled receptor GPR43, stimulating the production of GLP-1, a key regulator of insulin secretion. Several studies linked the ingestion of β-glucans with an improvement in glucose tolerance by increasing GLP-1 [10,11,12]. We performed a high-throughput static fecal fermentation study in which a set of fecal samples from healthy donors were incubated in the presence of HS-BG fiber. All the tested microbial communities were able to metabolize HS-BG fiber and we observed consistent production of SCFAs, independent of the initial human microbial composition, and increases in butyrate-producing microbial taxa (Figure 2 and Figure 3). Due to the limitations of static fecal fermentations, we evaluated the impact of HS-BG supplementation over 3 weeks on SCFA production in a dynamic, in vitro gut model (SHIME^®^) of the entire gastrointestinal tract. As expected, we observed that HS-BG fiber was not digested in the stomach/small intestinal vessels. It was fermented in the proximal and distal colon vessels, producing high amounts of SCFAs and increasing the relative abundance of butyrate-producing microbial species (Figure 4 and Figure 5). The SHIME^®^ results aligned with the observations in the static fecal fermentations, confirming that HS-BG fiber is a strong SCFA precursor, which, in humans, could stimulate GLP-1 and impact blood glucose dynamics. It is important to highlight that SCFA production was robustly consistent between gut fermentation models and very distant microbial communities. In addition, the production of SCFAs seems to have a restorative and anti-inflammatory impact on disrupted cell cultures (Figure 6 and Figure 7), which aligns with what was described for other β-glucans [12]. A disrupted and inflamed gut has been associated with the onset of diabetes [13], and it has been demonstrated that high fiber in a diet is one of the fastest strategies to strengthen the gut barrier [14].

While the impacts of long-chain β-glucans on human health have been well studied, the inability to deconvolute their intrinsic viscosity from their chemical structure has resulted in a large body of work linking viscosity to health outcomes and a lack of research looking into how non-viscosity attributes can influence host biology. One of the limiting factors for performing in vitro studies with β-glucan is the insolubility of large β-glucan fibers, which we have overcome through depolymerization. We acknowledge several limitations of the in vitro assays we performed since they do not fully model the complexity of biological systems found in vivo. Enzymatic assays such as α-glucosidase inhibition or glucose transporter inhibition assays represent valid screening tools, acting as the first step in evaluating the potential effect of a novel ingredient on key mechanisms associated with glycemic control. However, additional studies are needed to assess efficacy, safety, and dosing in humans. Similar limitations are found in microbiome-based experiments presented in this study, which only partially represent the complexity within the human gut environment. However, our multi-donor approach, which samples across a population of healthy donors, enables us to robustly assess the effects of HS-BG fiber on a general population.

We have demonstrated that HS-BG fiber may impact postprandial blood glucose control through microbiome-mediated butyrate production, which can lead to GLP-1 secretion through the biochemical inhibition of key carbohydrate digestive enzymes and glucose transporters. These results support, for the first time, that cereal β-glucans, independent of their polymerization degree or viscosity, can have profound impacts on host health. Furthermore, the reduced viscosity and increased solubility enable the incorporation of HS-BG fiber into many food matrices that meet the consumer’s demand for desirable taste and texture. This combination of functionality and palatability enables the formulation of food and beverage products that will empower consumers to better control their blood glucose levels through their diet.

## 5. Patents

HS-BG and its applications are subject to patent applications.

## Figures and Tables

**Table 1:** Inhibition of SGLT1 (*n* = 3) by different concentrations of HS-BG fiber. Assay buffer without fiber acts as negative control and phlorizin as positive control.

Compound	HS-BG Fiber Concentration [mg/mL]	Relative SGLT1 Inhibition (%) ± Standard Deviation
Assay buffer without fiber	0.0	0.0 ± 1.6
HS-BG fiber	2.1	6.0 ± 3.3
	6.8	12.0 ± 5.8
	9.8	17.3 ± 1.5
	12.7	23.3 ± 7.6
Phlorizin	100 (µM)	100.0 ± 0.3

## Data Availability

The original contributions presented in the study are included in the article, further inquiries can be directed to the corresponding author.
